# Membranes for Water and Wastewater Treatment

**DOI:** 10.3390/membranes11040295

**Published:** 2021-04-19

**Authors:** Asunción María Hidalgo, María Dolores Murcia

**Affiliations:** Departamento de Ingeniería Química, Facultad de Química, Univesidad de Murcia, 30100 Murcia, Spain

Water is a vital element for life and the environment. Water pollution has been documented as a contributor to a wide range of health problems. In recent years, water quality levels have suffered a great deterioration because of rapid social and economic development and because it is used to “dump” a wide range of pollutants.

This Special Issue entitled “Membranes for Water and Wastewater Treatment” contains featured research papers dealing with recent developments and advances on all the aspects related to membrane for water and wastewater treatment: membrane processes, combined processes (including one membrane step), modified membranes, new materials, and the possibility to reduce fouling and improve the efficiency of enhanced processes. 

The papers compiled in this Special Issue can be read as a response to the current needs and challenges in membrane development for water and wastewater treatment (Table 1). A total of 23 articles have been accepted; in total, 22 of them correspond to research articles in different fields, and one is a review paper. 

Half of the research articles correspond to concrete and practical applications of the use of membrane processes in different fields of the industry, with the aim of treating and conditioning water and wastewater. The studies reveal the treatment of industrial streams, mining, recycled paper industry, olive mill, urban wastewater, etc. Another important percentage of studies are related to the membrane modification processes with the aim of obtaining new materials with better performance in the separation processes, thus describing the use of membranes modified with chitosan, nanoparticles, and other organic compounds. This field also includes studies related to fouling and its modeling. Another field that is opening corresponds to the membranes of ion exchange resins and liquid membranes, and finally, the importance of the economic study to be able to predict the change of the membranes is also very interesting.

The revision paper carried out by Zhang et al. [1] about diffusion dialysis for acyl recovery from acidic waste solutions showed three important problems and directions for further improvements in anion exchange membranes (AEMs). The chemicals with high stability and alkalinity can be used as modifiers to prepare AEMs with improved acid recovery and stability. The materials with a size-sieving effect could be introduced into AEMs to enhance acid selectivity. Finally, the acidic functional groups, such as –COOH and –HSO_3_, have an excellent effect on the acid recovery of AEMs and could even overcome trade-off effects.

About 50% of the published works related to the applications in the field of the industry use nanofiltration membranes in the processes of separation and treatment of wastewater. Cristóvão et al. [2] tested the occurrence of the broad-spectrum fluoroquinolone antibiotics ciprofloxacin and levofloxacin in real wastewater effluents using a nanofiltration pilot-scale unit installed in the same sampling site of the WWTP. The results of a 24 h assay conducted at a constant pressure of 6 bar showed that the permeance was maintained and that a high removal of antibiotics, antibiotic resistance genes, and viral genomes can be expected with this treatment process. 

In the treatment of mine water, one of the main problems is the risk of crystallization of sparingly soluble salts on the membrane surface (scaling). Mitko et al. [3] studied a series of batch-mode nanofiltration experiments of the mine waters performed in a dead-end Sterlitech^®^ HP 4750X Stirred Cell. Based on the laboratory results, the concentration profiles of individual ions along the membrane length in a single-pass, industrial-scale nanofiltration (NF) unit was calculated, assuming the tanks-in-series flow model inside the membrane module. The dead-end experiments showed that the nanofiltration process may be safely operated even at 80% recovery of permeate.

The experiments carried by Marecka-Migacz et al. [4] using nanofiltration processes in the separation of aqueous solutions containing nitric salts of Zn, Cu, Fe, or Pb at various pH showed that it is possible to obtain the total volume membrane charge densities through mathematical modeling based on the Donnan–Steric partitioning model.

Hidalgo et al. [5] evaluated the performance of polyamide nanofiltration membrane on the removal of six different dyes. It has been proven that the chemical structure of the dyes has an important influence on the permeate fluxes and rejection coefficients obtained, these being the molecular volume and the length perpendicular to the maximum area the most relevant parameters. 

The feasibility of reverse osmosis (RO) for treating coking wastewaters from a steel manufacturing plant, rich in ammonium thiocyanate was assessed by Álvarez et al. [6]. DOW FILMTECTM SW30 membrane performance with synthetic and real thiocyanate-containing solutions was established at the laboratory and (onsite) pilot plant scale. No short-term fouling was observed, and the data followed the known solution–diffusion model and the film theory. 

Bottino et al. [7] used the integrated pressure-driven membrane processes for the treatment of olive mill wastewater (OMW). They consist of a first stage (microfiltration, MF) in which a porous multichannel ceramic membrane retains suspended materials and produces a clarified permeate for a second stage (reverse osmosis (RO)) in order to separate (and concentrate) dissolved substances from water, thus allowing the concentration of valuable products and produce water with low salinity, chemical oxygen demand (COD), and phytotoxicity.

Sousa et al. [8] investigated the optimization of the ultrafiltration (UF) process to remove colloidal substances from a paper mill’s treated effluent. The effects of four operating parameters in a UF system (transmembrane pressure (TMP), cross-flow velocity (CFV), temperature and molecular weight cutoff (MWCO)) on the average permeate flux (Jv), organic matter chemical oxygen demand (COD) rejection rate, and the cumulative flux decline (SFD) was investigated by robust experimental design using the Taguchi method. The results demonstrate the validity of the approach of using the Taguchi method and utility concept to obtain the optimal membrane conditions for the wastewater treatment using a reduced number of experiments.

Membrane bioreactors (MBRs) have a great potential for the treatment of municipal and industrial wastewater. The papers published in this Special Issue showed the relevance of the characterization of the activated sludge and the study of the fouling phenomena. Ding et al. [9] found that the membrane fouling rate of the anaerobic membrane bioreactor (AnMBR) at 25 °C was more severe than that at 35 °C. The membrane fouling trends were not consistent with the change in the concentration of soluble microbial product (SMP). On the other hand, Tabraiz et al. [10] investigated the status of acyl-homoserine lactone (AHL) in the sludge and biofilm of conventional AnMBR and the upflow anaerobic membrane bioreactor (UAnMBR), as well as in the sludge of a UASB reactor, all treating real sewage. Specifically, the work focuses on the relationship between the microbial community profile and the AHL detected in these membrane/sludge-based anaerobic systems, especially when they operate under extreme conditions (i.e., low temperatures). According to the authors, the molecules C10-HSL, C4-HSL, 3-oxo-C4-HSL, and C8-HSL are the main AHL present in anaerobic reactors (with or without membranes); these molecules require special attention in future work to further understand their role in biofilm formation/fouling and granulation.

In addition, other studies carried out show the need to model fouling phenomena and determine the mechanisms that influence these processes. Xu et al. [11] propose a semi-empirical multiple linear regression model to describe flux decline, incorporating the five fouling mechanisms (the first and second kinds of standard blocking, complete blocking, intermediate blocking, and cake filtration) based on the additivity of the permeate volume contributed by different coexisting mechanisms. On the other hand, Huang et al. [12] investigated the membrane fouling mechanism based on that combined models could provide theoretical supports to prevent and control UF fouling for surface water treatment.

The scaling and performance of flat sheet aquaporin FO membranes in the presence of calcium salts were examined by Omir et al. [13]. These authors found that the amount of sodium chloride (NaCl), saturation index, cross-flow velocity, and flow regime all play an important role in the scaling of aquaporin FO flat sheet membranes.

In the last decade, new materials and fabrication processes have been developed to improve performance in membrane synthesis and membrane-modification processes. The study conducted by Proner et al. [14] about modifying commercial ultrafiltration membranes to induce antifouling characteristics shows the relevance of investigating parameters such as the influence of membrane pore size and the polymer concentration used in modifying the solution. Other works show that it is possible to modify a thin-film composite nanofiltration membrane using a novel and facile method based on introducing Ca^2+^ in the heat posttreatment. Hand et al. investigated the introduction of Ca^2+^ induced in situ Ca^2+^-carboxyl intra-bridging, leading to the embedment of Ca^2+^ in the polyamide (PA) layer [15]. 

Zhou et al. [16] reported the use of a porous carbon nitride (C_3_N_4_) nanoparticle to potentially improve both the water flux and salt rejection of the state-of-the-art polyamide (PA) thin-film composite (TFC) membranes. Benefitting from the positive effects of C_3_N_4_, a more hydrophilic, more crumpled thin-film nanocomposite (TFN) membrane with a larger surface area and an increased cross-linking degree of PA layer was achieved.

Nady et al. [17] compared the efficiency of a conventional chlorination pretreatment with a novel modified low-fouling polyethersulfone (PES) ultrafiltration (UF) membrane in terms of bacteria attachment and membrane biofouling reduction. The results showed that the filtration of pretreated, inoculated seawater using the modified PES UF membrane without the prechlorination step maintained the highest initial flux (3.27 ± 0.13 m^3^·m^−2^·h^−1^) in the membrane, as well as having one and a half times higher water productivity than the unmodified membrane. 

The use of chitosan as a cross-linked agent to obtain new membranes has been reported in this Special Issue. Saiful et al. [18] developed a forward osmosis (FO) membrane from a mixture of chitosan and *Dioscorea hispida* starch, which was cross-linked using glutaraldehyde. The cross-linked chitosan/starch membrane was revealed to have high mechanical properties with an asymmetric structure. On the other hand, Nakayama et al. [19] prepared chitosan membranes by the casting method combined with alkali treatment. The molecular weight of chitosan and the alkali treatment influenced the water content and water permeability of the chitosan membranes. The water content increased as the NaOH concentration was increased from 1 to 5 mol/L. The water permeation flux of chitosan membranes with three different molecular weights increased linearly with the operating pressure and was highest for the membrane formed from chitosan with the lowest molecular weight. Membranes with a lower water content had a higher water flux. 

Among the advances in membrane preparation and modification, exchange ionic resins and liquid membranes have obtained special attention. Volkov et al. [20] investigated the cation-exchange membranes based on cross-linked sulfonated polystyrene (PS) grafted on polyethylene with an ion-exchange capacity of 2.5 mg-eq/g, while Erol et al. [21] reported the performance comparison of four commonly used cation exchange resins (Amberlite IR120 Na+, Amberlite IRP 69, Dowex MAC 3 H^+^, and Amberlite CG 50) and their influence on the current efficiency and selectivity for the removal of cations from a highly concentrated salt stream. The current efficiencies were high for all the resin types studied. Results also revealed that weak cation exchange resins favor the transport of the monovalent ion (Na^+^), while strong cation exchange resins either had no strong preference or preferred to transport the divalent ions (Ca^2+^ and Mg^2+^).

León et al. [22] studied the pertraction of Co(II) through novel supported liquid membranes prepared by ultrasound, using bis-2-ethylhexyl phosphoric acid as the carrier, sulfuric acid as stripping agent, and a counter-transport mechanism.

Finally, the economic study is very important to evaluate the effectiveness of applied processes. Bai et al. [23] studied the economic performance of the renovation via the net present value (NPV) method. The result reveals that the NPV of the renovation of the WWTP within the 20-year life cycle is CNY 72.51 million, and the overall investment cost can be recovered within the fourth year after the reoperation of the plant.

## Figures and Tables

**Table 1:** Summary of the detailed information of the publications in this Special Issue.

Numbers and Type of Articles	Fields	Industrial Process	Type of Membrane Process	Model	References
Review (1)	Diffusion dialysis	Acidic waste solution	Anion Exchange	-	[1]
		Antibiotics	NF	-	[2]
		Mine	NF	-	[3]
		Nitrate salts and heavy metals	NF	Donnan–Steric partitioning model	[4]
		Dyes	NF	Spiegler–Kedem–Katchalsky	[5]
		Steel	RO	Solution–Diffusion	[6]
		Olive mill	MF +RO	-	[7]
		Recycler paper and cardboard	UF	-	[8]
		Municipal wastewater	AnMBR	-	[9]
		Sewage	AnMBR	-	[10]
	Fouling		MF + UF	Semiempirical Multiple Linear Regression	[11]
		Surface water	UF	Combined models	[12]
		Aquaporin	FO	-	[13]
		Dopamine	UF	-	[14]
		Ca^2+^	NF/RO	-	[15]
		Chitosan+ alkali		-	[16]
		Chlorination pretreatment	UF	-	[17]
		Chitosan	FO	-	[18]
		Nanoparticles	RO	-	[19]
	Resins		Ion Exchange	-	[20,21]
	Liquid membrane		Liquid Membrane	-	[22]
	Economic study	WWT plant	UF	-	[23]
